# Electric Field Effect of the Plasma-Initiated Polymerization of Methyl Methacrylate: A Negatively Charged Long-Lived Radical

**DOI:** 10.3390/polym16111497

**Published:** 2024-05-24

**Authors:** Jiayu Rui, Siru Cheng, He Ren, Sheng Cui, Jian Huang

**Affiliations:** College of Materials Science and Engineering, Nanjing Tech University, No. 30 Puzhu Road (S), Nanjing 211816, China; ruijiayu1@163.com (J.R.); sirucheng2022@126.com (S.C.); 17612581005@163.com (H.R.); scui@njtech.edu.cn (S.C.)

**Keywords:** plasma-initiated polymerization, electric field, negatively charged radical, long-lived radical, one-electron transfer initiation

## Abstract

Plasma-initiated polymerization (PIP) is generally attributed to a radical process due to its inhibiting property. However, its unique polymerization behaviors like long-lived radical and solvent effect do not comply well with the traditional radical mechanism. Herein, the PIP of methyl methacrylate (MMA) was conducted in a high-voltage DC electric field to investigate the charged nature of its radicals. Consequently, the polymerization presented a preferential distribution of polymers at the anode but not the cathode, revealing the negatively charged nature of the growing radicals. An acceleration phenomenon, accompanied by the growth in molecular weights and the reduction in molecular weight distributions (*Ð*), was observed at the voltages above 16 kV, suggesting the dissociation of ion pairs of growing radicals. The PIP yielded PMMA with analogous chemical and steric structures to those of PMMA from traditional radical initiation, whether in the presence or absence of the external electric field. This work offers new insights into the PIP of vinyl monomers, wherein a one-electron transfer reaction is inferred to be involved in the monomer activation.

## 1. Introduction

Low-temperature plasma contains many active species, such as electrons, radicals, ions, photons and excited molecules, on the basis of which plasma is extensively applied in areas like surface treatment [1], environmental protection [2], agriculture [3], medicine [2,4], nanoscience [5], and catalysis [6], et al. Owing to the complexity of plasma discharge, however, the plasma technologies usually achieve only poor control in the chemistry of their products or present indefinite mechanisms [2,3,7,8,9]. Benefiting from a mild energy level, close to covalent bonds, low-temperature plasma is used as well in polymer preparation, e.g., plasma polymerization and plasma-initiated polymerization (PIP) [7,8,9]. While exposed in a sustained plasma discharge, vinyl monomers produce crosslinked polymers, generally with group structures different from those of the starting monomers. This process is referred to as plasma polymerization, in which the group destruction arising from plasma etching is generally inevitable [7,8,9]. Within the PIP, plasma discharge is merely imposed at the beginning to initiate vinyl monomers. The propagate reaction takes place via the addition of unsaturated bonds, as a conventional polymerization does. Therefore, the post-polymerization proceeds in the absence of plasma initiation, and yields linear polymers with considerably high molecular weights. Nevertheless, there still exist some questions about PIP, e.g., the long-lived radical and solvent effect, which have puzzled researchers up to the present [9,10].

Early in 1978, Osada et al. observed the PIP of vinyl monomers [10]. The polymerization was suggested to arise from a radical initiation, due to its inhibiting property of radical scavengers like 1,1-diphenyl-2-picrylhydrazyl (DPPH) and hydroquinone [11]. The other confirmation of the radical mechanism was its initiating capacities in the controlled radical polymerizations of ATRP, DT and RAFT, when appropriate mediating agents were applied [12,13,14]. However, there are some unique polymerization behaviors in PIP that cannot be well explained by traditional radical mechanisms. For instance, the post-polymerization could last as long as several days without extra plasma initiation, revealing the long-lived radical employed in the polymerization [10,15]. Using PIP, a block copolymerization could be achieved by sequentially adding the second vinyl monomer [15]. Moreover, the PIP usually exhibited a remarkable solvent effect, i.e., preferring to give a higher polymerization rate in an aqueous medium than in an organic one [11]. These phenomena appeared not only in the solution polymerization of PIP but in graft polymerization on polymer surfaces, when the polymerizations were initiated in the gaseous phase of frozen monomer solutions and directly on solid polymer surfaces, respectively [11,13]. The radical was formed irrespective of the complex components of plasma and the various organic precursors of monomers, solvents or polymer surfaces.

To comprehend these unusual phenomena, Osada et al. initially suggested the formation of a biradical intermediate in the plasma discharge of vinyl monomers [16]. Radicals were subsequently yielded via a slow decomposition of the intermediates. The sustained post-polymerization and the accompanied increase in molecular weights were ascribed to the reduced radical dimerization caused by the gel effect. However, this traditional radical could not account for the solvent effect usually occurring in the PIP. A few years later, they found that plasma-exposed N,N′-dimethylformamide (DMF) was able to reduce viologen compounds via a one-electron transfer reaction [11]. Noteworthy is that this plasma-exposed DMF could initiate vinyl monomers as a direct plasma discharge. They accordingly speculated that the radical formation was closely related to an electron–monomer interaction, whose activity strongly relied on solvent mediums. This conception, to some extent, is supported by the work by Go et al. that plasma discharge was rich in electrons and inclined to a reduction reaction [17]. Indeed, radical formation is more probable in a plasma discharge, because the electron energy (1–4 eV) in plasma is well matched with the bond energy of organic precursors. In contrast, ionization is difficult due to the high ionization energy (10 eV) of organic compounds. In a plasma atmosphere, the radical concentration should be 10^3^–10^5^ times higher than that of ions [7,8].

More recently, our group reported a novel controlled radical polymerization starting from solvated electrons [18]. The initiating system possessed higher molar conductivities and was in the state of less-paired solvated electrons. Interestingly, this polymerization exhibited the long-lived radical and solvent effect analogous to the case of PIP. Moreover, a remarkable electric field effect was observed in the polymerization, when polymers preferred to migrate towards the anode but not the cathode. In view of the one-electron transfer capability of solvated electrons, a negatively charged radical was proposed to be the growing species. It is noted that both PIP and solvated electron-initiated polymerization display similar unique polymerization behaviors and, more importantly, are all rich in electrons in their initiating systems. A one-electron transfer reaction is inferred to be involved in their monomer initiations. We, hence, conceive that PIP should show the charged nature of growing radicals like the solvated electron-initiated polymerization. Although some charged species have ever been envisioned to be associated with the growing species of PIP, little experimental evidence was provided to support these viewpoints [9,11]. This work aims to investigate the influence of an external electric field on the PIP of methyl methacrylate (MMA), in an effort to reveal the charged nature of its growing radicals. The polymerization behaviors in the electric field environment, including kinetics, molecular weights and chemical and steric conformations of PMMA, are evaluated in detail.

## 2. Materials and Methods

### 2.1. Materials

Hexamethylphosphoramide (HMPA), received from Nanjing Harper Pharmaceutical Company (Nanjing, China), was purified by vacuum distillation after dehydration using sodium pieces. Methyl methacrylate (MMA), purchased from Shanghai Lingfeng Chemical Reagent Company (Shanghai, China), was pre-dried by anhydrous sodium sulfate and distilled in a vacuum. Benzoyl peroxide (BPO) and N,N′-Dimethylaniline were obtained from Shanghai Lingfeng Chemical Reagent Company (Shanghai, China), and used as received.

### 2.2. Plasma-Initiated Polymerization under Electric Field

**Figure 1:**
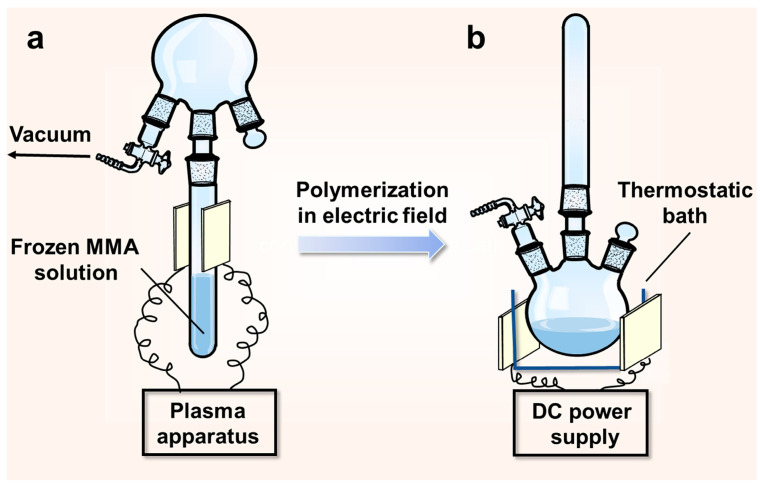
Experimental setup of the PIP. (**a**) The plasma initiation of the MMA solution; (**b**) the polymerization under a DC electric field.

The polymerization was performed in a three-neck flask equipped with a glass tube and a vacuum line, as seen in Figure 1. The reaction system was placed upside down and then loaded with 15 mL monomer solution (40 vol% MMA in HMPA) in the glass tube (Figure 1a). The monomer solution was degassed by repeated freezing and thawing to remove oxygen. After a refreezing of the solution by liquid nitrogen, the reaction system was evacuated to 10 Pa. The glass tube containing the frozen monomer solution was inserted between two capacitively coupled electrodes that were attached to the plasma apparatus with an RF power of 13.56 MHz. Plasma discharge was ignited in the vapor phase of the frozen monomer solution, lasting for 60 s at the power of 60 W, after which the frozen solution was melted and mixed instantly with the atmosphere of the reaction system. The reaction system was turned upwards, as seen in Figure 1b, and placed with its flask between the two parallel-plate electrodes connected to a high-voltage DC power supply (DW-P303-1ACF0, from Dong Wen High Voltage Company, Tianjin, China). The plasma-initiated polymerization (PIP) proceeded in the flask at the constant temperature of 50 °C, under electric field voltages of 0–28 kV. Mechanical stirring was absent throughout the polymerization so that we could observe the possible polymer migration in the electric field environment. After prescribed periods of time, the polymer solutions located in both electrode regions were drawn out by syringes, and poured into the precipitation agent of alcohol. Polymeric products were acquired via drying in a vacuum at the temperature of 80 °C.

### 2.3. Traditional Radical Polymerization under Electric Field

A three-neck flask was applied to conduct the traditional radical polymerization (RP). A monomer solution including 10 mL MMA and 20 mL HMPA, mixed with 0.056 g BPO and 0.06 mL N,N′-Dimethylaniline, was poured into the flask and purged with Ar to remove oxygen. The polymerization proceeded at the same temperature of 50 °C, as the PIP. The polar solvent of HMPA was re-employed in the RP to distinguish its possible effect in the electric field environment. The utilization of the external electric field and sample collection were identical to those described above for the PIP.

### 2.4. Characterizations

The molecular weights and distributions of polymers were measured on the gel permeation chromatography (GPC) system, equipped with a 515 HPLC pump from Waters (Milford, MA, USA), a digital refractometer of Optilab T-rEX from Wyatt (Santa Barbara, CA, USA), a multi-angle laser light scattering instrument of DAWN-HELEOS-II MALS from Wyatt (Santa Barbara, CA, USA), and a liquid chromatography column (MZ-Gel SDplus LS Linear 10 µm, Column 300 mm × 8 mm, Mzanalysentechnik, Tübingen, Germany). The mobile phase of tetrahydrofuran ran at a speed of 1.00 mL/min and a column temperature of 35 °C. Fourier transform infrared spectroscopy (FT-IR) was determined on the spectrophotometer NEXUS 670 from Nicolet (Madison, WI, USA), using a potassium bromide disk and the wavenumber range from 4000 to 400 cm^−1^. Spectra were recorded with 64 scans in the resolution of 4 cm^−1^. Nuclear magnetic resonance analyses (^1^H NMR) were carried out on an AVANCE AV 500 spectrometer of Bruker (Fallanden, Switzerland), with the frequency of 500 MHz and deuterochloroform as the solvent.

## 3. Results and Discussion

### 3.1. Polymer Migration and Negatively Charged Radical

**Figure 2:**
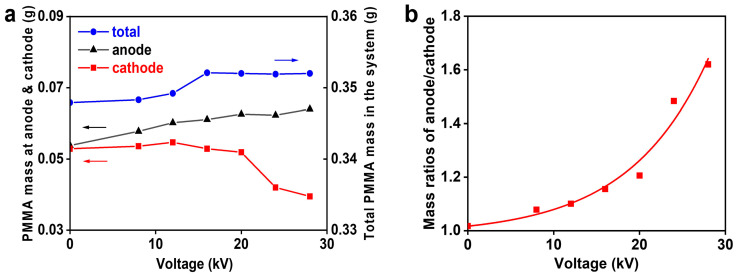
Dependence of the PIP on electric field voltages with the polymerization time of 8 h. (**a**) The PMMA amounts distributed in both electrode regions; (**b**) the PMMA mass ratios of anode/cathode.

The PIP of MMA was conducted under the high-voltage DC electric field while its initiation was performed without the electric field. To avoid a direct electrode reaction with reactants, both anode and cathode were put outside the flask wall. Because we aimed to investigate the possible polymer migration towards electrodes, mechanical stirring was absent throughout the polymerization. Upon the completion of the polymerization, the polymer solutions located in both electrode regions were taken out, respectively. As expected, the polymer distributions at both electrodes failed to exhibit a visible discrepancy when at zero voltage. Nevertheless, they grew apart gradually with the increase in electric field voltages, as seen in Figure 2a. This phenomenon indicates the preference of polymer migration towards the anode; contrarily, the polymers were repelled by the cathode under the electric field. This polymer migration was more clearly manifested by the enlarged polymer mass ratios of anode/cathode at higher electric field voltages, as seen in Figure 2b. These results, to some extent, reveal the negatively charged nature of the growing radicals responsible for the PIP, because of the typical electroneutrality of PMMA chains. The electroneutrality of PMMA is further verified by its poor mass deviation at both electrodes, when PMMA was prepared via a traditional radical initiator under the same electric field environment (seeing below). In low-temperature plasma, electrons are quickly accelerated owing to their small mass, while ions remain near the ambient temperature. Electrons can, hence, gain a higher thermal flow velocity than ions. Consequently, a species in plasma can easily develop a negative potential due to electron attachment [7,8]. This plasma process supports the negatively charged nature of radicals induced by plasma. Owing to the coulombic repulsion between mutual charges, radical dimerization is greatly hindered, which can endow the radicals in PIP with a long life. In addition to the polymer distributions at both electrodes, the total polymer amounts in the whole reaction system are also shown in Figure 2a. From the total polymer amounts, an acceleration phenomenon can be seen at the voltages above 16 kV (Figure 2a and Figure S1). This rate increase suggests an ion pair conformation of the growing radicals, since a dissociated ion pair is helpful for rapid polymerization [19]. An ion pair separation might have been made by the high voltages above 16 kV, which would have led to the high reactivity of the growing radicals.

It is noted that the polymer migration in the PIP behaved like it had taken place in the solvated electron-initiated polymerization under the electric field [18]. An almost identical mass ratio curve to that of PIP (Figure 2b) was seen in the latter polymerization. In addition to the electric field effect, however, both polymerizations presented other common features, such as a long-lived radical, solvent effect (Figure S2), and a low propagating constant (*k_p_* = 10^−2^ − 10^−3^ L · mol^−1^ · s^−1^) [11,12,13,16,18], which do not comply with the traditional radical mechanism. In light of the electric field effect and the inhibiting property of radical scavengers, a negatively charged radical was suggested to be responsible for the solvated electron-initiated polymerization. Benefiting from the coulombic repulsion between the negatively charged radicals, the polymerization could maintain a higher radical concentration (~10^−3^ mol/L) and, hence, presented a higher rate close to that of RP, despite its low *k_p_* value [18]. Moreover, it is worth mentioning that these unique polymerization behaviors also appeared in the polymerization initiated by samarium diiodide (SmI_2_) [18], a typical one-electron transfer agent [20].

**Figure 3:**
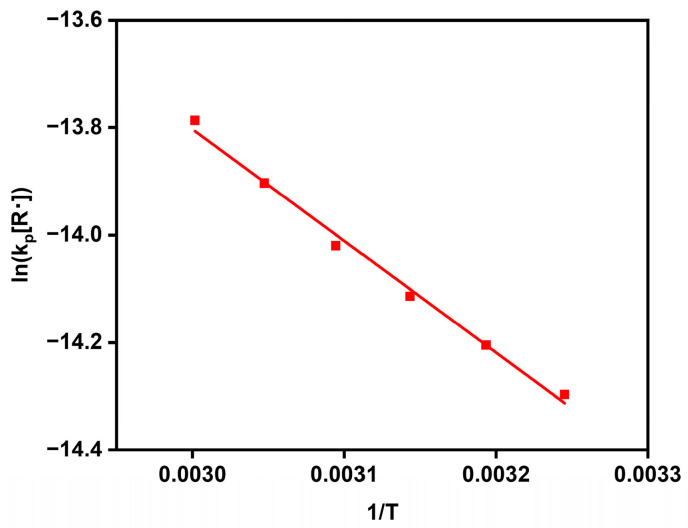
Arrhenius line of PMMA in the PIP at temperatures from 35 to 60 °C.

The temperature dependence of the PIP was examined and is shown in Figure 3. The apparent activation energy of 17.3 kJ/mol was obtained from the Arrhenius line. In view of the long-lived feature of growing radicals, this value could largely be attributed to the propagating activation energy (*E_p_*) of the PIP. Such an *E_p_* result, however, is close to that of PMMA in a RP [21], regardless of the negatively charged nature of the growing radicals in the PIP. In addition, this *E_p_* value is similar to that of PMMA detected in the solvated electron-initiated polymerization [18]. Therefore, the PIP gave a normal *E_p_* value but a low propagating constant (*k_p_*), like the case that occurred in the solvated electron-initiated polymerization. This conflict in *k_p_* and *E_p_* values suggests a small pre-exponential factor (Arrhenius equation) in the PIP. The radical formation may heavily depend on certain intermediates produced in the PIP.

Considering the essential characteristic of the PIP and solvated electron-initiated polymerization, their initiating systems are all rich in electrons or prone to donate electrons [17,22]. They may apply an analogous mechanism in monomer activation, i.e., involving a one-electron transfer reaction towards monomer molecules. Nonetheless, more work is needed to investigate their polymerization behaviors and reveal their probable relations. On the basis of the negatively charged nature of growing radicals, the monomer initiation of the PIP is roughly envisioned, as seen in Equation (1):
(1)
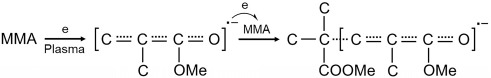

As a conjugated and electron-deficient monomer, MMA inclines to receive an electron from plasma, and yields an intermediate loaded by a negative charge. The monomer addition takes place via a radical process, which should be closely associated with a derivative of the intermediate. The propagating reaction is inferred to proceed along with a successive electron migration from the intermediate to the next monomer unit. Because of the charged property of the intermediate, its reaction activity and the polymerization rate should be greatly influenced by the solvents used in the PIP.

### 3.2. Molecular Weight and Its Distribution

**Figure 4:**
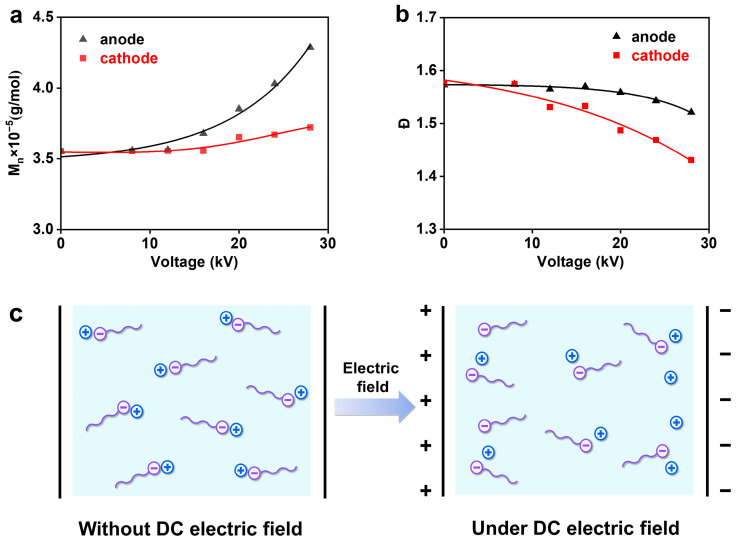
Dependence of the PIP on electric field voltages with the polymerization time of 8 h. (**a**) The molecular weights of PMMA located at both electrodes; (**b**) the molecular weight distributions (*Ð*) of PMMA located at both electrodes; (**c**) schematic illustration of the migration phenomena of PMMA chains (anions at termini) and counterions (cations) towards both electrodes in the PIP conducted under the DC electric field.

In view of the negatively charged nature of growing radicals, the influence of an external electric field on the molecular weights of polymers was investigated. The molecular weights of PMMA, located in anode and cathode regions, were detected and are shown in Figure 4a. Both molecular weight results increased with the enhancement of voltages, but the anodic ones were more susceptible to the electric field voltage. Resembling the phenomenon occurring in polymerization rates (Figure 2a), the molecular weights rose obviously above the voltage threshold of 16 kV. This increase in molecular weights, to some degree, agrees with the suggestion that the growing radicals apply an ion pair conformation, because a dissociated ion pair in a strong electric field is highly reactive and apt to produce polymers with high molecular weights. Thus, the higher susceptibility of the molecular weights at the anode to the voltages can be interpreted by the ion pair conformation. As indicated by the uneven distribution of polymers at both electrodes (Figure 2a), the PMMA chains tend to migrate towards the anode, owing to the electronegativity of their termini, but the counterions (cations) prefer to go in the opposite direction. Excess anions (PMMA termini) and cations are, hence, enriched at the anode and cathode, respectively, to offset the external electric field, as is schematically shown in Figure 4c. Being deficient in counterions, the less-paired radicals at the anode, hence, become highly reactive and are beneficial to a rapid growth of molecular weights. In contrast, surrounded by extra counterions, the more-paired radicals at the cathode merely show relatively little activity that is unfavorable to a rapid growth of molecular weights. In this sense, the pairing status of radicals should also contribute the rate discrepancy appearing at both electrodes (Figure 2a), except for the mentioned effect of polymer migration.

With the increase in molecular weights, however, the molecular weight distributions (*Ð*) were decreased at higher voltages, as seen in Figure 4b. A more obvious reduction in *Ð* values was seen at the cathode relative to the case at the anode. The lowest *Ð* value of 1.43 was reached at the cathode under the highest voltage of 28 kV. This declination in *Ð* values is explained by the relieved radical dimerization in the electric field environment. As discussed above about Figure 4c, excess anions and cations tend to accumulate at the anode and cathode in the electric field environment, respectively. The growing radicals, hence, display somewhat net-negative charges at the anode due to the lack of counterions, while they show somewhat net-positive charges at the cathode because of the enrichment of counterions. The radical dimerization is considerably inhibited by the enhanced coulombic repulsion, which is helpful for attaining a low *Ð* value. In the cathode region, however, the radical dimerization can be further depressed by the steric hindrance coming from extra counterions, which gives rise to the much lower *Ð* values of PMMA.

### 3.3. Long-Lived Radicals in Electric Field

**Figure 5:**
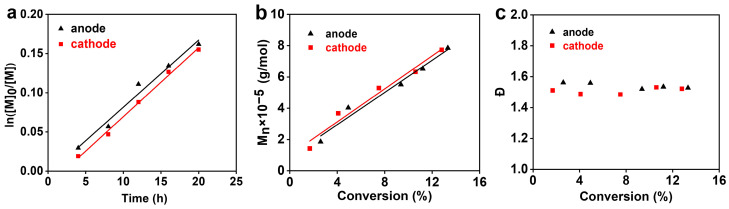
Polymerization kinetics of PMMA in the PIP under the electric field voltage of 20 kV. (**a**) Ln([M]_0_/[M]); (**b**) molecular weight; (**c**) molecular weight distribution (*Ð*).

Due to a negatively charged radical, the PIP exhibited notable behaviors of polymerization rate and molecular weight in the DC electric field. Its long-lived radicals in the electric field environment is worth further evaluation. The polymerization kinetics was investigated using the voltage of 20 kV (Figure 5 and Figure S3), a value higher than the voltage threshold of 16 kV, when the ion pairs of the growing radicals were thought to be in an electric field-separated state. Both kinetic lines of the anode and cathode presented linear growth, as shown in Figure 5a, indicating the constant radical concentrations in the polymerization. The radical life was maintained, like the case in the PIP without the electric field (Figure S2). The long-lived radicals were verified as well by the proportional increase in molecular weights with conversions, as seen in Figure 5b. On this basis, predictable molecular weights can be reached by the PIP. At the same polymerization time, the anode always gave larger polymer amounts and higher molecular weights than those of the cathode (Figure 5a and Table 1), agreeing with the inclination of the polymer migration and the higher activity of radicals at the anode. The *Ð* values of both electrodes are shown in Figure 5c, when the *Ð* values remained approximately stable because of their discrepancies less than 0.08 for all polymers. It is envisioned that a controlled radical polymerization could be developed based on the PIP, like the one-electron transfer initiation by solvated electrons [18], if some side reaction and the *Ð* values of the PIP could be further depressed by adopting appropriate chemical or physical conditions.

### 3.4. Traditional Radical Polymerization in Electric Field

**Figure 6:**
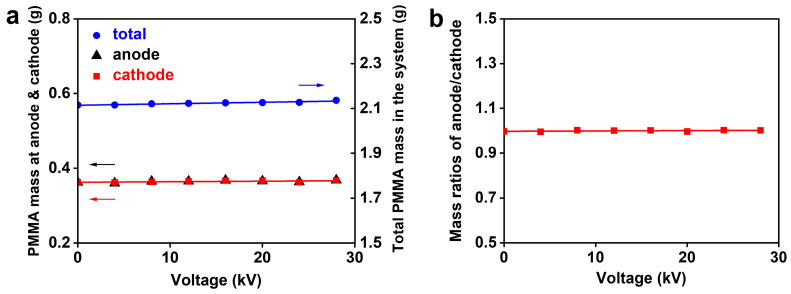
Dependence of the RP on electric field voltages. (**a**) The PMMA amounts distributed in both electrode regions (the data of anode and cathode are almost identical); (**b**) the PMMA mass ratios of anode/cathode.

To further confirm the charged nature of the growing radicals in the PIP, the traditional radical polymerization (RP) of MMA was conducted in the electric field. In the RP, the same reaction conditions were utilized as in PIP, except for a redox initiator. The polar solvent of HMPA was re-employed to distinguish its possible role in the electric field effect. Figure 6 shows the mass distributions of PMMA at both electrodes. The polymers gave almost level and overlapped mass lines at both electrodes with the enhancement of voltages, as exhibited in Figure 6a. These results indicate the uniform distribution of PMMA in the reaction system, which is more clearly illustrated by the nearly constant mass ratios of 1 at various voltages (Figure 6b). This poor influence of the external electric field indicates the electroneutrality of PMMA chains, from which the charged property of growing radicals in the PIP could be reasonably proposed. Additionally, the polar solvent of HMPA is proved to be unrelated to the origin of the electric field effect occurring in the PIP.

### 3.5. FT-IR and ^1^H NMR Analyses

**Figure 7:**
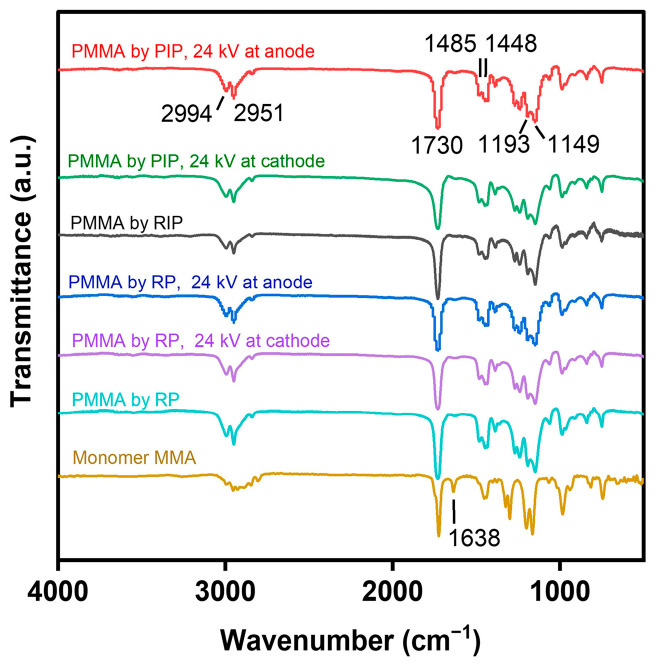
FT-IR spectra of PMMA prepared by PIP and RP under the electric field.

**Figure 8:**
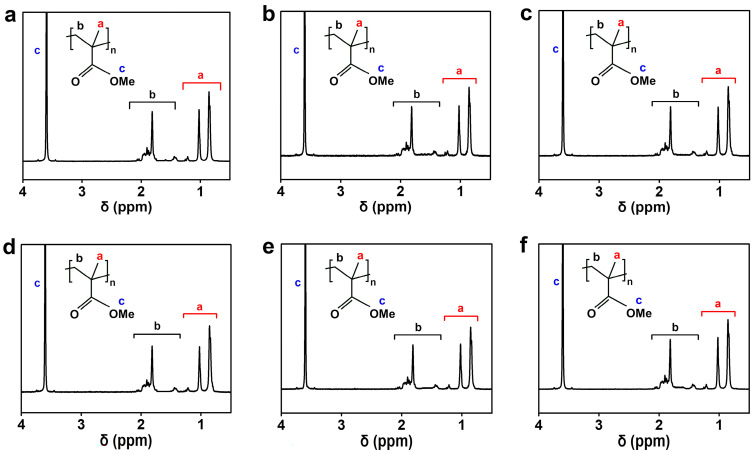
^1^H NMR spectra of PMMA prepared by the PIP and RP under the electric field. (**a**) PIP, 24 kV at anode; (**b**) PIP, 24 kV at cathode; (**c**) PIP, without electric field; (**d**) RP, 24 kV at anode; (**e**) RP, 24 kV at cathode; (**f**) RP, without electric field.

The chemical constitutions of polymeric products, prepared by the PIP in the electric field, were analyzed by FT-IR and ^1^H NMR, as shown in Figure 7, Figure 8, Figures S4 and S5. In the FT-IR spectra of Figure 7 and Figure S4, the polymers exhibited the antisymmetric and symmetric stretching vibrations of C-H at 2994 and 2951 cm^−1^, the stretching vibration of C=O at 1730 cm^−1^, the deformation vibrations of C-H at 1485 and 1448 cm^−1^, and the antisymmetric and symmetric stretching vibrations of C-O at 1193 and 1149 cm^−1^, respectively, which agree with the corresponding characteristic bands of PMMA from the radical initiation [23,24]. The absence of the alkene signal at 1638 cm^−1^ in the polymers, by comparison with MMA, proves that the addition reaction took place via the C=C bond instead of the C=O bond of MMA [24], irrespective of the negative charges loaded on the radicals of PIP. In the ^1^H NMR spectra of Figure 8 and Figure S5, the polymers also presented analogous results to the PMMA from radical initiation. The characteristic signals emerging at 3.60, 2.04–1.44, and 1.26–0.84 ppm are attributed to the -OCH_3_, -CH_2_-, and -CH_3_ groups of PMMA, respectively [25]. These analyses reveal that PIP yielded polymers with chemical constitutions similar to the PMMA produced by RP, although the polymerization started from a negatively charged radical and was conducted in a DC electric field.

The tacticity of the polymers was calculated according to the split peaks of the α-methyl group at 1.26–0.84 ppm [18], as shown in Figure 8, Figure S5 and Table 2. The PIP in the electric field gave similar tacticity data to those by the RP, when they both inclined to a syndiotactic addition of MMA. In the RP of MMA, the preference for a syndiotactic placement can be interpreted by the α-methyl repulsion between growing chains and incoming monomers [19], even if a traditional radical generally leads to an atactic addition due to the planar sp^2^ configuration. In the solvated electron-initiated polymerization of MMA, however, a slight increase of isotactic triads was seen under the electric field [18]. The decreased sensibility of the tacticity to the external electric field in the PIP could be ascribed to the larger counterions in the ion pairs of the growing radicals, although the counterions are not well defined in a plasma discharge. By comparison with the smaller counterion of Na^+^ in the solvated electron-initiated polymerization, the larger counterion in the PIP is relatively insusceptible to the electric field because of its low charge density. In addition to the cases of PIP, the effect of the electric field on the PMMA structures of the RP is also shown in Figure 7 and Figure 8. The polymers gave almost the same results in terms of constitutions and tacticity whether in the presence or absence of the electric field, because of the electroneutrality of PMMA.

## 4. Conclusions

This paper deals with the PIP of MMA that was performed in a DC electric field. The polymers preferred to migrate towards the anode but not the cathode when polymerized in the electric field environment. This inclination suggests the negatively charged nature of growing radicals responsible for the PIP, which was verified by the electroneutrality of PMMA chains in the RP under the same electric field. The polymerization was accelerated at the voltages above the threshold of 16 kV, accompanied by a concurrent increase in molecular weights and a decrease in *Ð* values. These phenomena can reasonably be ascribed to the electric field-dissociated ion pairs of growing radicals. The polymerization maintained the long-lived property of the growing radicals in the electric field, by which the molecular weights presented a proportional growth with conversions. The PIP produced PMMA with similar chemical constitutions and tacticity to those by the RP, irrespective of the negatively charged nature of radicals and the adoption of an external electric field. Considering the richness of electrons in plasma discharge, a one-electron transfer reaction is inferred to be involved in the monomer activation of PIP.

This work offers new insights into the monomer initiation of PIP, which is speculated to be closely related to other initiations involving one-electron transfer reactions, like solvated electrons and SmI_2_, because of their similar unusual polymerization behaviors. The exploration of the electric field effect and the corresponding proposition about the one-electron transfer initiation in the PIP are potential ways to illuminate other plasma processes whose mechanisms are not quite sure nowadays.

## Figures and Tables

**Table 1:** Conversions and molecular weights of PMMA at both electrodes, in the PIP with various polymerization times under the electric field voltage of 20 kV *.

Polymerization Time (h)	Anode		Cathode	
	Conversion (%)	Molecular Weight (×10^−5^)	Conversion (%)	Molecular Weight (×10^−5^)
4	2.6	1.852	1.7	1.423
8	4.9	4.030	4.1	3.671
12	9.4	5.514	7.5	5.286
16	11.2	6.521	10.6	6.332
20	13.3	7.849	12.8	7.728

* The molecular weights and conversions are from the results shown in Figure 5b.

**Table 2:** Tacticity of PMMA from the PIP and RP, with and without the DC electric field.

Polymerization	DC Voltage (kV)	Electrode	mm (%)	mr (%)	rr (%)
PIP	24	anode	5.5	37.4	57.1
	24	cathode	5.1	36.9	58.0
	16	anode	5.6	37.5	57.9
	16	cathode	4.9	36.3	58.7
	—	—	4.6	37.7	57.7
RP	24	anode	5.6	36.5	58.0
	24	cathode	4.7	36.8	58.5
	—	—	4.7	37.3	58.0

## Data Availability

Date are contained within the article and Supplementary Materials.

## Supplementary Materials

The following supporting information can be downloaded at https://www.mdpi.com/article/10.3390/polym16111497/s1, Figure S1: The acceleration phenomenon of the PIP occurring at the electric field voltages above 16 kV; Figure S2: Polymerization kinetics of PMMA in the PIP conducted in bulk and aqueous medium (emulsion); Figure S3: Polymerization kinetics of PMMA in the whole reaction system of the PIP that was conducted at the electric field voltage of 20 kV; Figure S4: FT-IR spectra of PMMA prepared by PIP under the electric field; Figure S5: 1H NMR spectra of PMMA prepared by PIP under the electric field. (a) PIP, 16 kV at anode; (b) PIP, 16 kV at cathode.
