# Supplementary material for: Electric Field Effect of the Plasma-Initiated Polymerization of Methyl Methacrylate: A Negatively Charged Long-Lived Radical
Source: Polymers (Basel). 2024 May 24;16(11):1497. doi: 10.3390/polym16111497 (PMC11174972; doi:10.3390/polym16111497)
Supplement: Supplementary file 1 [file polymers-16-01497-s001.zip › polymers-3011478-Supplementary File.pdf]

Electric Field Effect of the Plasma-Initiated Polymerization of Methyl Methacrylate: A Negatively Charged Long-Lived Radical

Jiayu Rui, Siru Cheng, He Ren, Sheng Cui, Jian Huang*

College of Materials Science and Engineering, Nanjing Tech University, No. 30 Puzhu Road (S), Nanjing 211816, P.R. China.

* Correspondence: jhuang@njtech.edu.cn

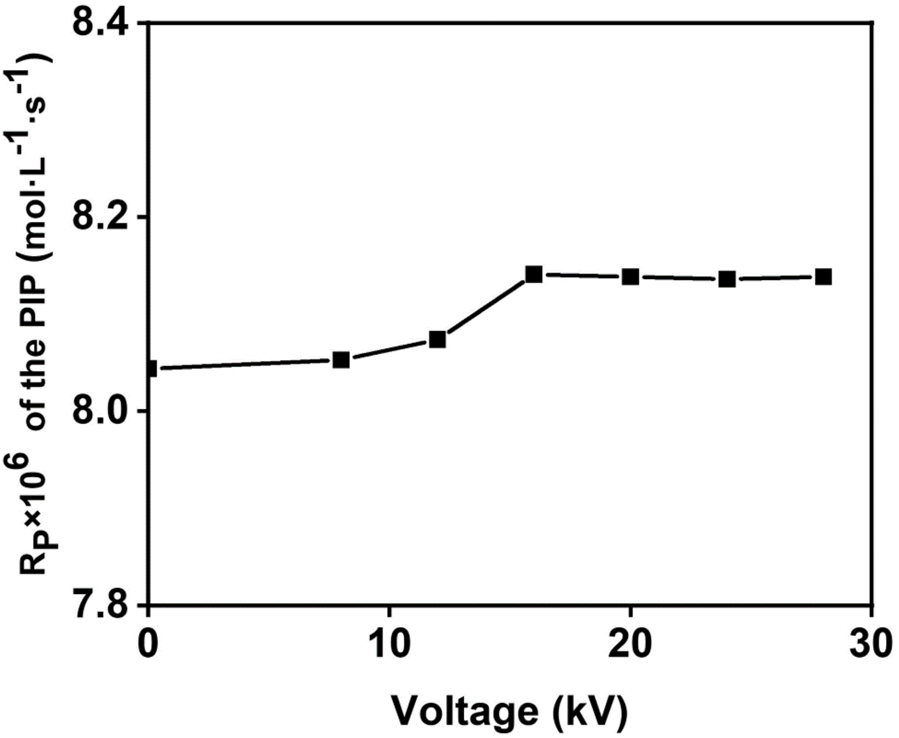

Figure S1. The acceleration phenomenon of the PIP occurring at the electric-field voltages above 16 kV. The polymerization rates (R_p) were calculated from the total polymer amounts in the whole reaction system shown in Figure 2.

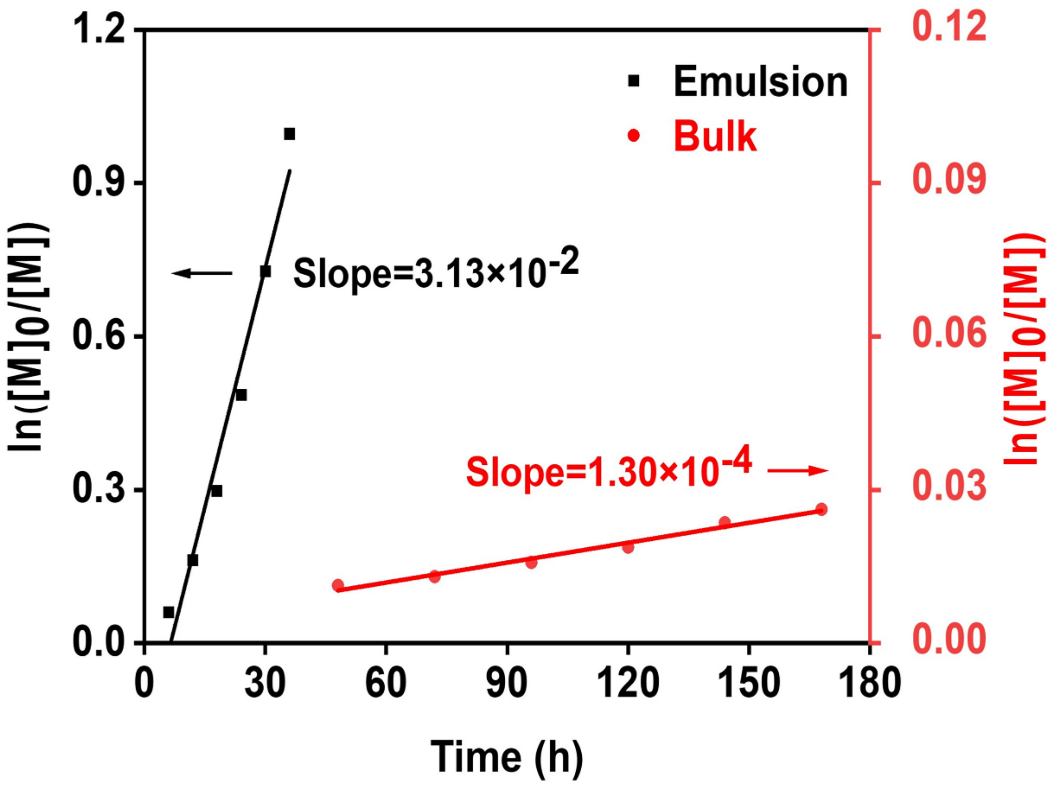

Figure S2. Polymerization kinetics of PMMA in the PIP conducted in bulk and aqueous medium (emulsion).

The PIP of MMA shows the long-lived nature of growing radicals and remarkable solvent effect. The polymerization was conducted in bulk and aqueous medium, using the plasma power of 90 W for 60 s at the polymerization temperature of 35 ºC. Limited by the less solubility of MMA in water, the polymerization in aqueous medium applied the emulsion method with the emulsifier concentration of sodium dodecyl benzene sulfonate at 8.0×10^{-3} mol/L. Both bulk and emulsion polymerizations gave substantially linear relations, indicating the long-lived radicals in the PIPs. However, the emulsion polymerization showed the rate 240 times higher than that of the bulk polymerization, indicating the remarkable solvent effect taking place in the PIP.

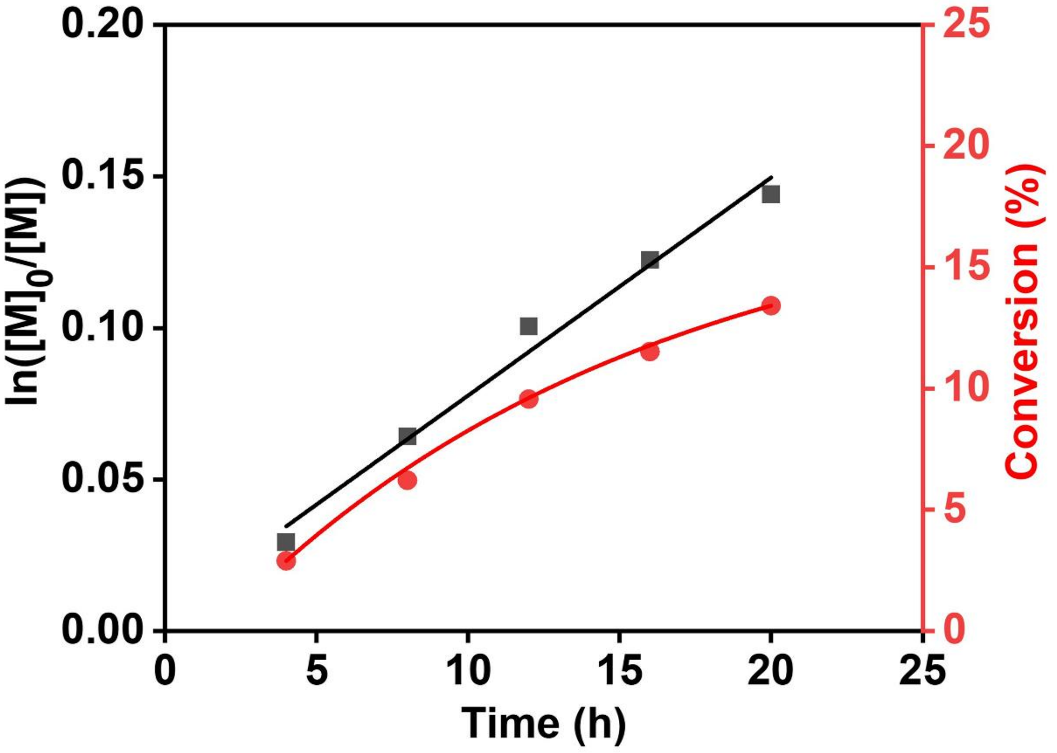

Figure S3. Polymerization kinetics of PMMA in the whole reaction system of the PIP that was conducted at the electric-field voltage of 20 kV.

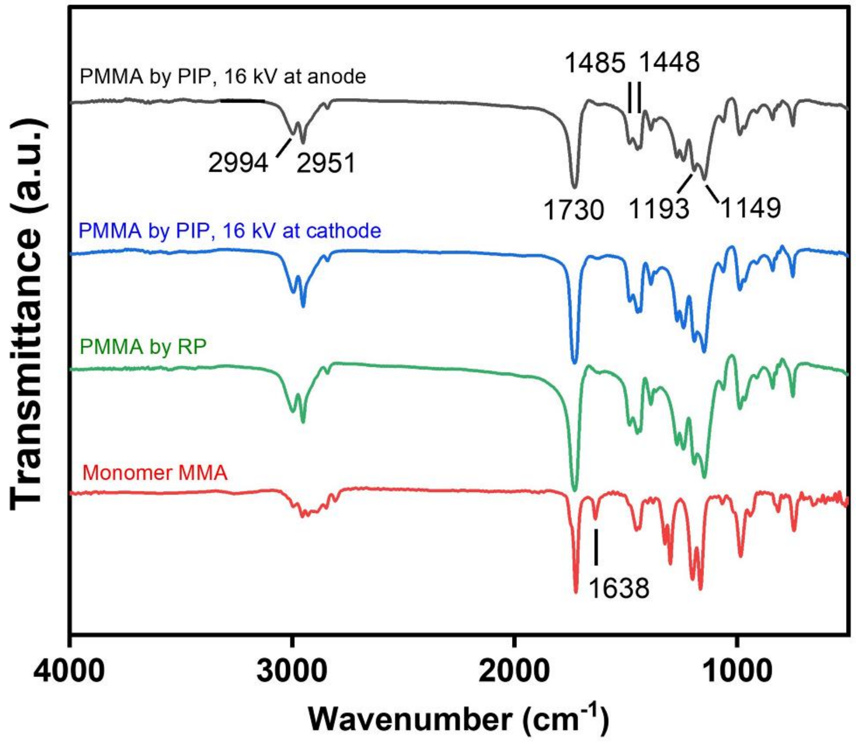

Figure S4. FT-IR spectra of PMMA prepared by the PIP under the electric

field.

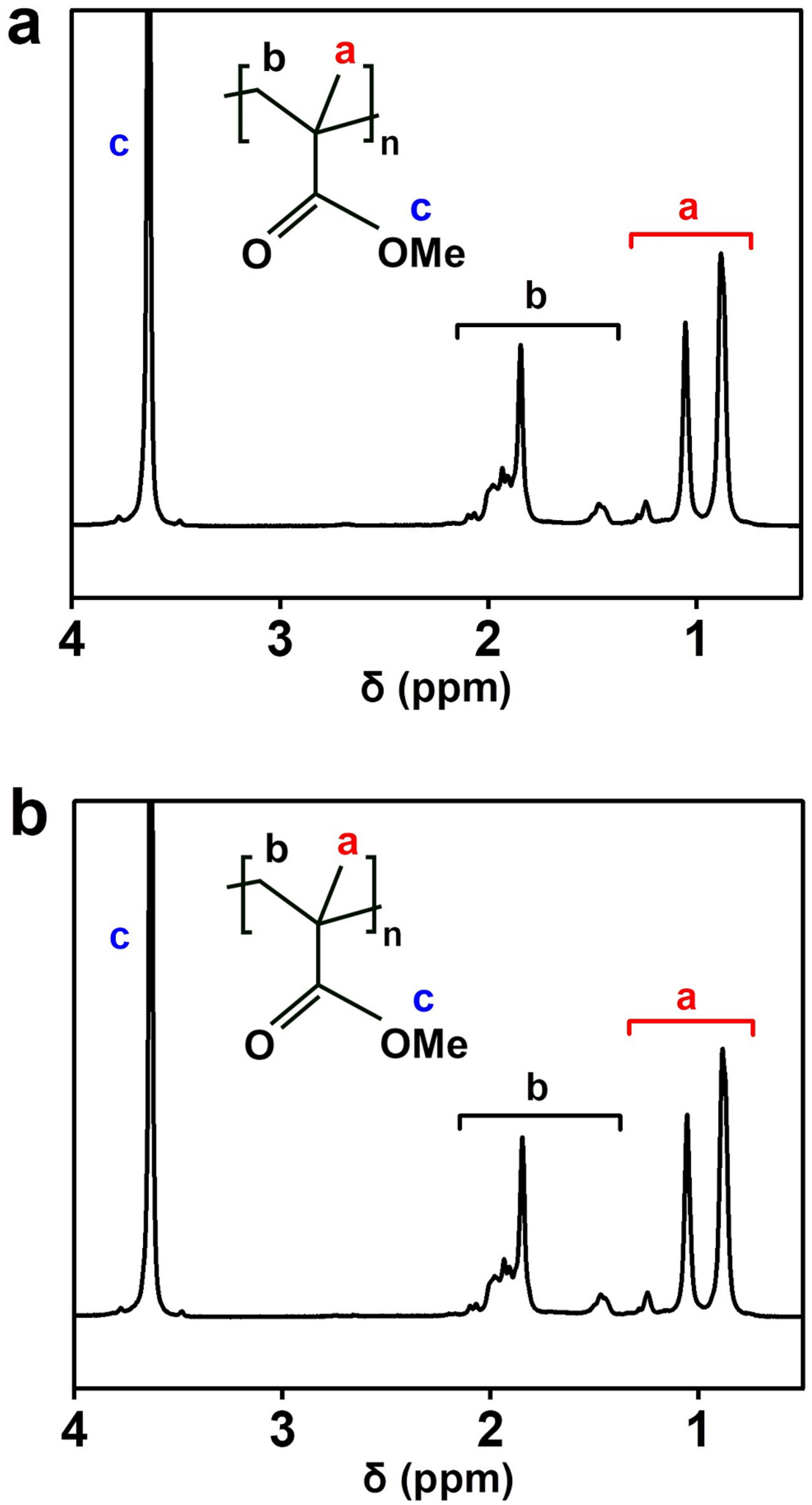

Figure S5. ^{1}H NMR spectra of PMMA prepared by the PIP under the electric field. (a) PIP, 16 kV at anode; (b) PIP, 16 kV at cathode.
